# Breaking the silence on femicide: How women challenge epistemic injustice and male violence

**DOI:** 10.1111/1468-4446.12968

**Published:** 2022-07-10

**Authors:** Baris Cayli Messina

**Affiliations:** ^1^ School of Social and Political Sciences University of Lincoln Lincoln UK

**Keywords:** digital ethnography, digital space, epistemic injustice, femicide, women movement

## Abstract

Digital space has provided an important platform for women by enabling them to defy religious and patriarchal values while rendering their demands more visible in the public sphere. By analyzing the stories of 3349 murdered women, consulting 57 activist‐published materials, studying 37 protest‐focused videos, and using digital ethnography, this article explores Turkish women's struggles against femicide. I propose the emancipatory and democratizing counterpublics as an analytical concept to demonstrate how women challenge epistemic injustice and male violence. To this end, I investigate the struggles of women by studying their use of digital space as a means of breaking the silence on femicide, creating data, disseminating knowledge, and seeking justice. This article highlights the essential role of new media technologies in empowering vulnerable groups through the generation of new forms of knowledge, the formation of collective memory, and the elimination of epistemic injustice in opposition to the ruling authorities. The present study contributes to our knowledge of the sociology of epistemic injustice by demonstrating how digital space plays a limited but critical role in the efforts of activists living under authoritarian regimes to defend their fundamental rights to survive and prevent femicide, which has a devastating impact on the lives of millions of women.

## 1 INTRODUCTION

Pınar Gültekin, a 27‐year‐old Mula University School of Economics student in Turkey, was abducted by Cemal Metin Avcı on July 16, 2020. Her corpse was discovered by authorities on July 21, 2020 in a remote location around 30 miles from her home (Birgun, 2021). It was found that the killer had bought gasoline to burn Gültekin's corpse and then poured concrete over her body. According to Gültekin's parents' account, Avcı was an aggressive stalker who never stopped harassing their daughter after she refused his advances (Haberler, 2020). As the details of the murder were revealed on television, many people, including important politicians and personalities from the country's popular media, expressed their outrage (Sozcu, 2020a). Gültekin's family was also supported by a number of Turkey's leading feminist groups, who launched an online campaign on social media with the hashtag #ChallengeAccepted and uploaded black and white selfies on Twitter (The Guardian, 2020). They wanted to draw attention to “femicide”—the murder of a woman by a man because of her gender. The internet campaign quickly spread around the globe, and Hollywood actors Nicole Kidman, Kate Hudson, and Jessica Biel joined the campaign by tweeting the hashtag, denouncing femicide, and eulogizing Gültekin (Sozcu, 2020b). With the support of local feminist organizations, collective resistance was expressed on digital platforms worldwide.

I began this paper with Gültekin's tragic murder to demonstrate how collective resistance through the use of digital space is critical for demonstrating solidarity in the face of trauma. With the support of local feminist organizations, collective resistance was expressed on digital platforms worldwide. According to Lukose (2018, p. 47), the surge in feminist resistance movements elicits “a politics of location as a frame for understanding the multiplicity of feminisms around the world.” The politics of location also matter to the feminist movement in Turkey. Those efforts have gained particular importance in a country where conservative politics and religious discourse have shaped people's lives for the last 2 decades. With the rise of the conservative AK Party's rule in 2003, competitive authoritarian politics has dramatically influenced social, political, and cultural life (Cayli et al., 2018; Esen & Gumuscu, 2016). In competitive authoritarian regimes, formal democratic institutions are the primary means of acquiring and exerting political authority. However, incumbents break those norms often and to such a degree that the system fails to fulfill traditional democratic basic criteria (Levitsky & Way, 2002, p. 52).

Recent scholarship has pointed to the pivotal role that social movements play in reducing gender‐based violence (Daby & Moseley, 2021; Huang, 2021; Miura, 2021; Ruiz, 2019; Shin, 2021; Storer & Rodriguez, 2020; Vindhya & Lingam, 2019). However, we still know very little about how digital space can promote the rights of vulnerable groups and influence the ways in which knowledge is received and conveyed under authoritarian regimes, particularly when it comes to politically polarizing issues reflecting the governing authorities' social and cultural norms, such as femicide. The present article aims to fill this gap by focusing on feminist mobilization against femicide in Turkey. The surge in the number of femicide cases and the brutal violence against women torment the lives of millions in the country. The public visibility of femicide cases has significantly increased thanks to the efforts of feminist activists. For example, the *We Will Stop Femicide Platform* ^1^ (“the Platform”) was established in 2010 in Istanbul by feminist activists who opened new branches across the country. Gülsüm Kav, the leader of the Platform, wrote an article in 2014 after Prime Minister Erdoğan criticized feminist activists who opposed femicide and asserted that women's primary role was motherhood: “Erdoğan cannot target and discriminate against feminists. He tries the politics of polarization for women, and this time he tries to divide women by saying, ‘we could not explain it to feminists, we will continue with those who understand us’. The best answer to Erdoğan can be given by women through fighting together shoulder to shoulder. The more he tries to divide, the more women unite. In addition, these women will hold Erdoğan to account if a feminist woman gets hurt”.

I focus on femicide and women's resistance in Turkey in order to tease out how women's actions undermine authority, limiting their rights, prompting me to question the role of epistemic injustice and its severe consequences for vulnerable groups living under authoritarian regimes. Epistemic injustice refers to knowledge forms that result in unfair, wrongful, and unjust treatment by influencing people's modes of behavior, participation, and communication, resulting in “silence, invisibility, and inaudibility (or distorted presence or representation); having one's meanings or contributions systematically distorted, misheard, or misrepresented” (Kidd et al., 2017, p. 1).

Through an analysis of the Platform's activities, publications, protests, and use of digital space, I argue that the Turkish feminist movement strives not only to prevent femicide but also to address epistemic injustice by creating a digital archive of femicide cases, disseminating knowledge, and politicizing the public sphere to effectively challenge the ruling authority. In this regard, activists' use of digital space provides new perspectives on how digital ethnography can be an instrumental discipline to explore power hierarchies and social conflicts in authoritarian regimes. The activists use digital space to expose the femicide and discrimination faced by the LGBTQ+ community, refugees, Kurdish women, and other ethnic minorities. Their search for justice has united heterogeneous and vulnerable groups from different segments of society in their endeavor to archive personal details involving women's murders. In doing so, they aim to inform the public, mobilize people, intervene in the policy‐making process, and defy the social and cultural norms of authoritarian regimes in the country.

(i) focus on two critical aspects in Turkish women's quest to create counterpublics: emancipation and (ii) democratization. In this study, I use the term “emancipation” to refer to overcoming impediments to self‐realization and fighting human exploitation against social, political, and legal restrictions. I employ democratization in the framework of democratizing the public realm by incorporating the voices of victims and survivors in order to influence public discourse and bring justice to vulnerable groups' lives. I introduce the concept of emancipatory and democratizing counterpublics to demonstrate that Turkish women's struggle to create counterpublics and prevent femicide is not only emancipatory but also democratizing, as they generate new data, disseminate it, explain the causes of femicide to the public, and provide a platform for vulnerable groups to make their demands accessible to everyone via digital space.

Using the emancipatory and democratizing counterpublics and deconstructing the struggle of women in digital space, I demonstrate how Turkish women's struggles against femicide have expanded across multiple power domains, including governmental actors, patriarchal cultural norms, and a knowledge system that restricts women's emancipation. In this context, digital space enables the development of practices and modes of intervention that result in the formulation of strategies to challenge the ruling authority, whose policies and standards exacerbate epistemic injustice. The expansion of epistemic injustice both poses risks to the emancipation of women and the democratization of the public sphere. Through the use of digital space, Turkish women have been able to preserve and share knowledge, calling into question the silence, denial, and complicity surrounding femicide. They have engaged in a dialectical relationship to broaden their resistance to hegemonic authority by using digital space and archiving femicide. This has enabled activists to create new knowledge, make victims' stories visible and establish new social networks of solidarity. In the following sections, I argue that the efforts of Turkish women to subvert hegemonic authority make them a counter‐hegemonic force, and their struggle for survival is a manifestation of emancipatory and democratizing counterpublics.

This study consists of five sections. In the first section, I provide a theoretical background related to the creation of counterpublics and methodological boundaries in researching epistemic injustice. Second, I introduce the methods used to collect the data. Third, I examine how women challenge epistemic injustice and male violence by using digital space and politicizing the public sphere. Fourth, I propose a framework of the public sphere of conflict explaining (a) the rationale for epistemic injustice and (b) the role of emancipatory and democratizing counterpublics to eliminate epistemic injustice based on the examined case study. Finally, I conclude with remarks on the role of digital ethnography for vulnerable groups and highlight the limitations and theoretical contribution of this study to the sociology of epistemic injustice.

## 2 CREATING COUNTERPUBLICS: METHODOLOGICAL BOUNDARIES IN RESEARCHING EPISTEMIC INJUSTICE

The term “counterpublics” arose out of debates about the public sphere in 1989, following the publication of Jürgen Habermas' *The Structural Transformation of the Public Sphere* (Fattal, 2018; Habermas, 1989). Public events and occasions, as opposed to individual or exclusive gatherings, are those that are open to all, according to Habermas (1989, p. 1). This “public sphere” is a domain of our social existence in which public opinion can be formed, and all private citizens are permitted to contribute to public opinion formation, which in turn shapes the discursive nature of politics (Habermas, 1989, pp. 138–140). Fraser criticizes Habermas's public sphere theory, claiming that subordinated groups that assert counterdiscourses are “subaltern counterpublics” (1990, p. 67). Fraser (1992, p. 128) pointed out that counterpublics aim to transform the public sphere “where members of subordinated social groups invent and circulate counter discourses to formulate oppositional interpretations of their identities, interests, and needs.” Fraser's argument is especially important considering the role of hierarchical power relationships in creating disadvantages for oppressed groups whose members lack epistemological tools to shape public opinion and challenge dominant public discourse. This results in the prevention of oppressed people from representing themselves in the public domain (Murray & Durrham, 2019, p. 5).

Discrimination against women in their daily lives paves the way for femicide. When government authorities normalize discrimination against women by assigning them the responsibilities and characteristics that promote and support patriarchy, their discourse can pose serious risks for women. This form of patriarchal knowledge and the values propagated by authorities reinforce an epistemic injustice that needs to be deconstructed to reveal its destructive outcomes and achieve gender equality. Epistemic injustice in this case study is inextricably linked to the activists’ efforts to create new data, learn new forms of knowledge, and share information in order to mobilize more people through digital space and tools. However, the activists' struggles are more than just a fight against femicide. Women are discriminated against on a daily basis, whether it is walking down a street late at night or being harassed and belittled in the workplace. Femicide is the most extreme consequence of this discrimination against women. This situation necessitates evaluating the role of digital space and social mobilization critically and drawing attention to methodological opportunities and limitations in the construction of counterpublics against those who dominate the public via political, legal, and media channels.

In her influential text, “Can the Subaltern Speak?,” Spivak (1988) describes epistemic violence as the consequence of silencing marginalized voices and, in that way, destroying their systems of knowledge, traditions, and beliefs. Epistemic violence thus becomes “the remotely orchestrated, far‐flung, and heterogeneous project to constitute the colonial subject as Other” (Spivak, 1988, p. 271). However, epistemic violence has a broader impact and is not confined to colonialist discourse. The most visible effect of epistemic violence is its repercussions in the public sphere. Fraser (1992, p. 110) defined the public sphere as the arena in which citizens gather to debate common issues. Authoritarian rule becomes more durable when oppressed individuals are unable to explain the violence and injustice they face. Authoritarian states restrict the space and means for citizens to contest their legitimacy (Klein & Lee, 2019; Torpey, 1998). Citizens can generate counterarguments against the state's discourse and policies, making the formation of counterpublics independent of the state's authoritarian role. However, even during the formation of counterpublics, oppositional actors can be directly influenced by the same policies and discourses imposed by state authority. Thus, despite the fact that counterpublic formations seek to limit the capacity of authoritarian state actors, the response of state authorities to such pressure from counterpublic actors stimulates punitive and repressive measures against the citizens who form counterpublics (Norderson, 2017). This conflict in the public realm inhibits citizens' access to epistemic tools since they have limited space and resources under the surveillance of authoritarian governments.

We can trace the origins of counterpublics back to the mobilization motivations of actors who were dissatisfied with the policies of the dominant authority (Cayli, 2013). This brings us to the concept of perceived injustice, which refers to groups that oppose the authority that governs public space, and their opposition serves as a catalyst for the formation of counterpublics (Salter, 2013). To be aware of such an injustice against oneself, however, we require epistemological tools that crystallize both the causes and consequences of the injustice that torments our lives (Cayli, 2016a, 2016b; Hookway, 2010; Lee, 2021). Fricker (2007) compellingly demonstrates how testimonial injustice—the devaluation or elimination of one's testimony—and hermeneutical injustice—the lack of resources/knowledge to interpret one's own experience create epistemic injustice. Moreover, different manifestations and practices of epistemic injustice can manifest themselves beyond linguistic expression (Dotson, 2011).

Participants and public debate are impacted by online discursive politics as it enables feminist activists to develop new strategies for engaging with and utilizing emerging communication platforms (Barker‐Plummer & Barker‐Plummer, 2017). Digital space incentivizes activists by providing them with tools to use physical space so they can express their concerns. For example, in India, women's experiences at physical protest locations are inextricably linked to the development of digital feminist networks that promote translocal relationships and maintain the movement via online activism (Bhatia, 2021, p. 1). Moghadam (2013, p. 8) succinctly pointed out that “the internet and especially social‐networking media came to be seen as significant new mobilizing technologies, helping to create ‘virtual communities’ or connect various movements, networks, and individuals for collective action framed by a collective identity.” Activists in Turkey launched the Monument Counter because it was difficult to get transparent data on violence against women from the Ministry of Justice, the Ministry of Family and Social Policy, and the General Directorate for Security in Turkey. Thus, a more reliable source was created by using digital methods and recording each femicide case. To be sure, documenting these murders was a painful and potentially traumatic process for activists. Nonetheless, it posed a challenge to epistemic injustice by obtaining information about women's murders that had been concealed by state institutions and using digital space to disseminate information about murdered women in order to raise public awareness and memorialize those who had been mistreated by political and judicial authorities.

In this respect, the Monument Counter^2^ (Anıt Sayaç in Turkish), manifests both epistemic injustice and the resistance against it. The Monument Counter is an online internet monument managed by Platform members that registers the murders of Turkish women by men and digitally narrates the stories of victimized women. The Monument Counter publishes photographs of murdered women alongside narratives elucidating the epistemic injustices women face. Numerous victims lack knowledge and resources; those living in villages, in particular, are unaware of how to respond to or avoid the murderers' threats and violence. Along with highlighting challenges to epistemic injustice, the Monument Counter serves as a model to eliminate epistemic injustice by utilizing digital space to reach the greatest number of women possible, from villages to cities. This method informs women about their rights and provides them with free services by professional volunteers ranging from shelter and accompaniment to the police station for drafting complaints to legal assistance.

The political opportunity structure focuses on how social movements not only challenge political institutions, but also interact with them in a variety of settings that determine the structure and degree of the challenge as well as its success probability (della Porta, 2013). Movements that show evidence of success, such as placing their causes in the national policy discourse or influencing public policy, are more likely to inspire countermovements (Meyer & Staggenborg, 1996, p. 1635). In this way, political opportunities are shaped by movements and countermovements, and the evolution of pertinent public policies is contingent upon the competition between conflicting actors (Mayer & Staggenborg, 1996). Coalitions with elites can provide significant acceleration to the relevant group if its members are united around broad goals and benefit from political opportunities and environmental factors (Staggenborg, 2010, p. 319). Meyer and Minkoff (2004, p. 1484) claimed that issue‐specific models in the political opportunity structure have better explanatory power than system‐wide dimensions. In addition, they asserted that elite support was crucial for exerting pressure on public officials and achieving the goals of the US civil rights movement (Meyer & Minkoff, 2004, p. 1482).

Digital space is not always utilized to improve women's rights; it is often used for sexism, posing risks to women (Banet‐Weiser & Miltner, 2016; Jane, 2014). However, examining the effective use of digital space to generate new knowledge compels us to reconsider the method by which women employ their ideas to generate new forms of knowledge and counterpublics in the digital realm. Collective solidarity, as Littler and Rottenberg (2020) persuasively argue, is critical for expanding feminist resistance and combating injustice. From this vantage point, archiving women's murders enables Turkish feminist activists to mediate this process of learning during their resistance, thereby granting them the capacity and authority to generate new data and strengthen their solidarity. Turkish feminist activists, on the other hand, not only mediate this learning experience, but also actively oppose a hegemonic authority that subjects them to patriarchal and religious norms. Their struggle for the generation of new data by using digital platforms also elicits methodological challenges in exposing epistemic injustice and the necessity of expanding the reach of their arguments and policies on femicide.

Corradi et al. (2016) emphasize the importance of a holistic and multidisciplinary approach to the study of femicide. More specifically, Dawson and Carrigan's (2020) comprehensive research explains how femicide differs from other types of homicide. They argue that creating reliable data is a primary motive for guiding research that saves women's lives. The work of feminist activists in Turkey is in line with these important assertions when they use digital space to archive the murder of women by men and disseminate the reasons for femicide. This generates reliable data that can be shared with the public and mobilizes more people on behalf of their cause. Turkish women directly fight against a competing vision that portrays them as subservient to hegemonic political authority's religious and patriarchal moral codes. Turkish women actively resist political authority and patriarchal norms by building a strong social network structure and mobilizing other women across the country through digital channels. From this point of view, the present case study attempts to show how digital space plays a role as an instrumental platform to record women's suffering and publicly make visible the injustices to which they are subjected. In this regard, creating counterpublics takes on particular significance when women in urban and rural areas face obstacles in seeking help to protect themselves from violence through the use of online channels, especially when they are under their abusers' surveillance.

Social movement actors must recognize the formidable role of media technologies and materials that they employ to interact with each other and historicize them so they can contextualize social movement actors in relation to media technologies (Mattoni, 2017, p. 494). Media technologies provide social movement activists with fertile ground for practicing their convictions and resisting the ruling authorities. McLean (2020) argued that the digital has created spaces for feminist activity, but at a cost; individuals and organizations are using digital technologies to reproduce and challenge sexist and misogynistic behaviors and systems. While the instruments that enable digital feminist activism are technically worldwide, they are not generally accessible, and so there are limitations and variations in terms of how successfully people use them. As a result, the digital space creates a platform for both risks and opportunities. To seize those opportunities, we need to develop methods for effectively utilizing digital space to challenge epistemic injustice through the generation of new data and mobilization of people.

Brooker and Meyer (2019, p. 253) pointed out that to fully understand the evolution, dynamics, and potential effects of protest movements, we must determine the possible coalition structures and their influence. Successful and long‐lasting mobilization requires organizing, engaging, and coordinating at least somewhat diverse interests. The use of digital space and the mobilization of women contribute to preventing femicide and resisting epistemic injustice by seeking allies and mobilizing people to influence policies and save the lives of women effectively. Hence, women's resistance may help persuade the public about the role of dysfunctional legal systems and governmental institutions in femicide. By collecting new data and educating the general public about femicide, a platform for counterpublics is formed. This transforms the fight against femicide into one for equality and justice for all vulnerable groups.

## 3 METHODS

Digital ethnography provides online data that is difficult to access through traditional methods (Murthy, 2008; Underberg & Zorn, 2014). It also offers an important source of dissemination through digital communication, especially when communicating effectively with vulnerable groups whose rights are constrained and whose voices are silenced. As Kavanaugh and Maratea (2019, p. 17) argue rightly, “studying digital communication can benefit from drawing more expressly on visual and counter visual approaches to examine practises of discourse containment, with an eye toward immanent critique”. The internet provides a kind of “cyber‐shelter” for marginalized groups that are unable to maintain physical locations owing to fear of discrimination, violence, and persecution—the internet enables those who confront difficulties to articulate the causes of their suffering and inequity (McLean, 2014). This is a result of how information technology has altered our ability to connect with others and think about ourselves and our identities (Hillier & Harrison, 2007).

**FIGURE 1:**
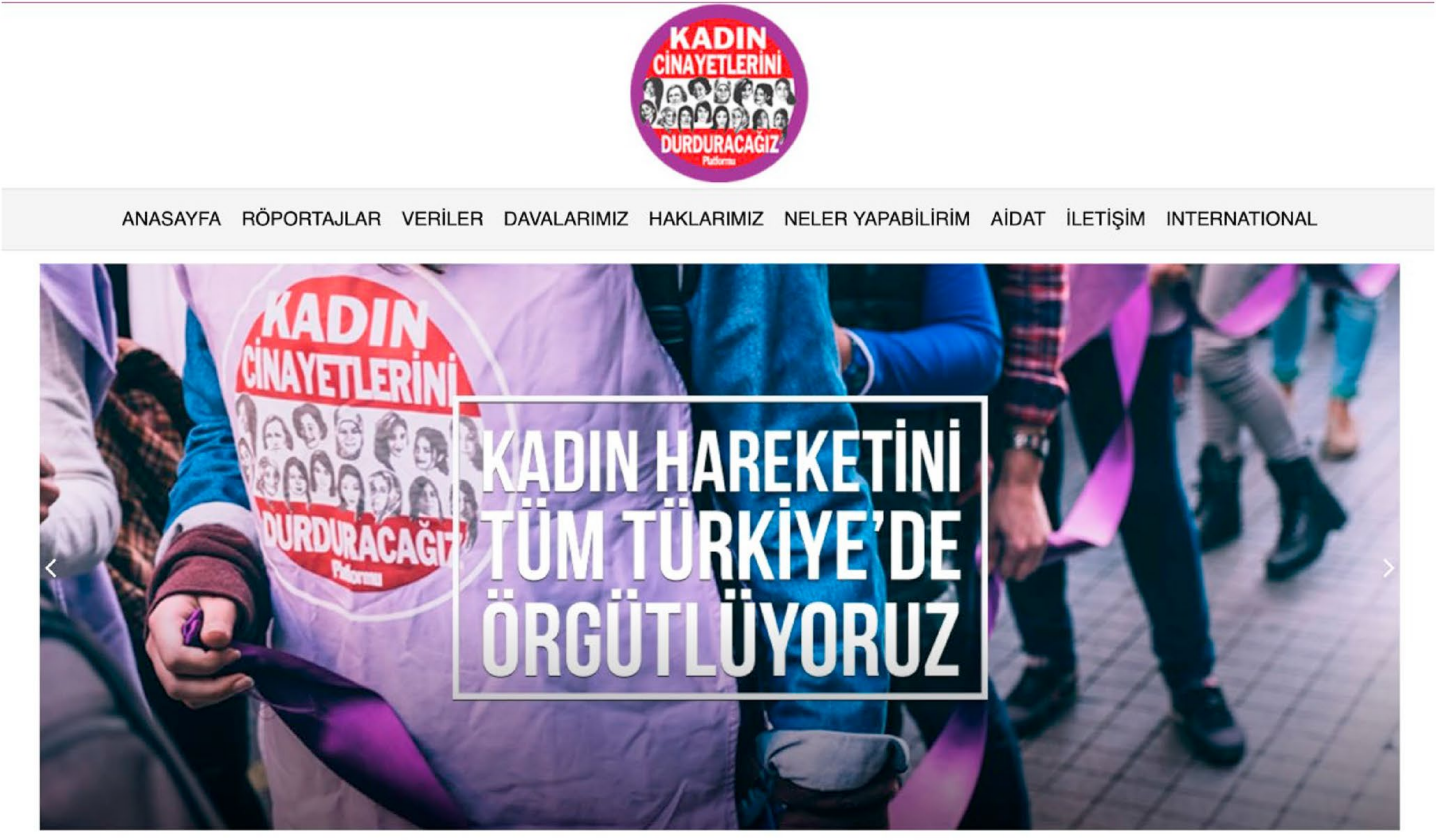
The message of the landing page of the Platform: “We are organizing the women movement in all over Turkey”

Through three primary instruments, the Platform's members utilize digital space to facilitate counterpublics: (i) the collection of the stories of murdered women in order to create a digital memorial that mobilizes people by appealing to their emotions; (ii) the organization of virtual events and the use of visual materials to list their demands and specify concrete policy changes they seek in current legal and political institutions to prevent femicide; and (iii) more effective communication with both platform members in dozens of cities via email and video conference, as well as the use of the same tools for survivors who are threatened with violence or death. I dug into their digital realm to discover how their resistance has grown stronger since they began using digital tools and networks to prevent femicide. Considering epistemic injustice and the activists' use of digital space, I used digital ethnography to analyze the Platform's website as a methodological approach to explore the complex social and cultural stigmatization of women and their struggle against femicide (see Figure 1).

**FIGURE 2:**
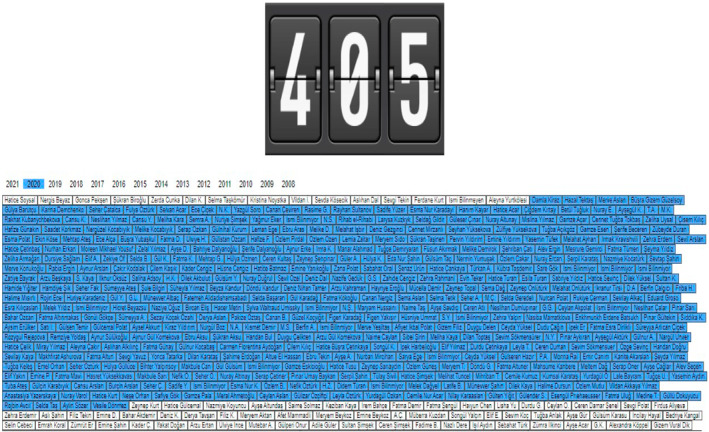
The image shows the number of women, 405, and their names who were murdered by men in 2020

I also consulted the Monument Counter, which publishes the collected data from national and local media outlets. Administrators receive information from hundreds of members across the country. The Monument Counter website classifies each femicide case by registering the victim's name and listing those names by year (see Figure 2).

**FIGURE 3:**
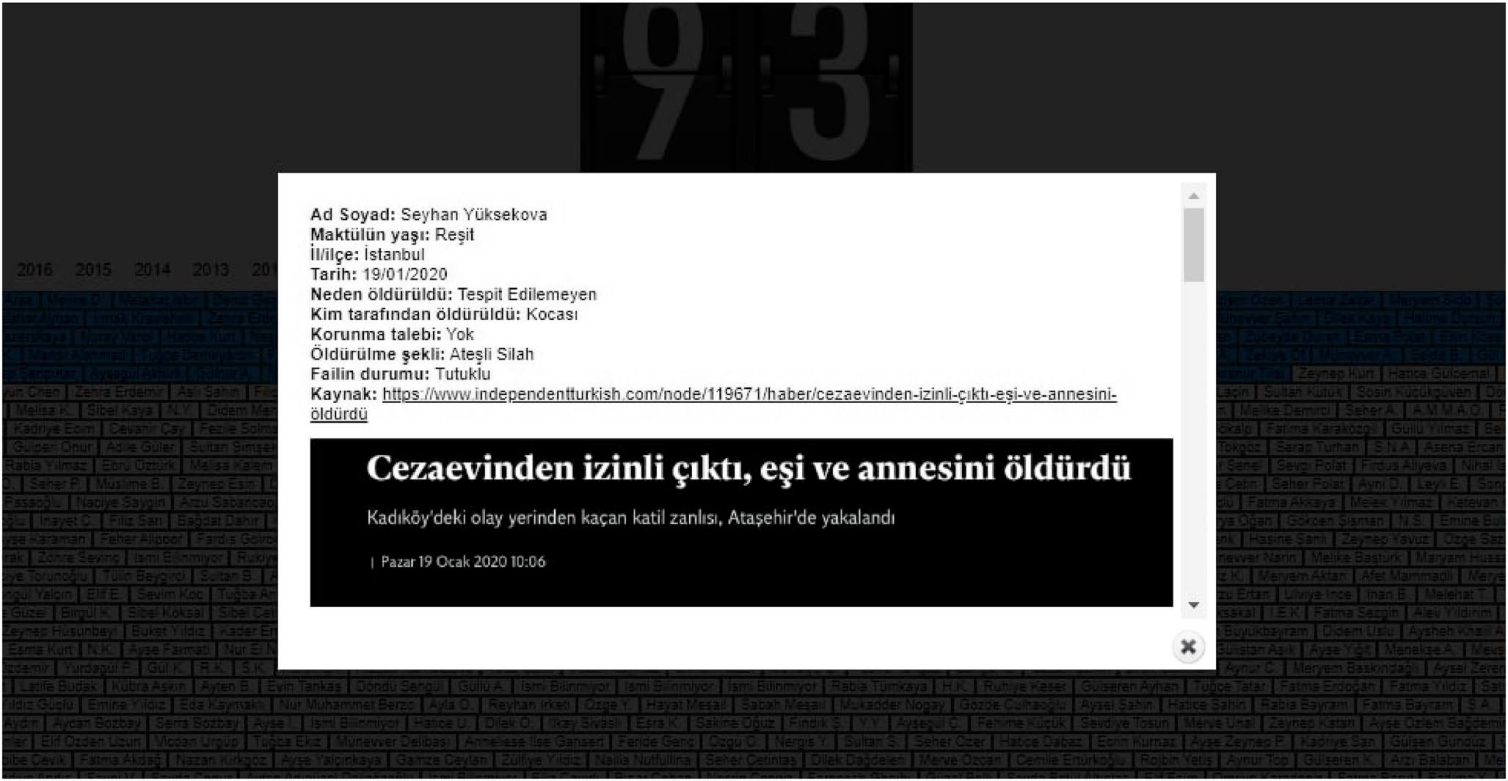
With a click of a murdered woman, Seyhan Yuksekova, a new window opens up and the visitor to the website can gain information about the femicide case, offender, and source of information

Clicking on each name opens a new webpage containing information on each femicide case (see Figure 3).

The website provides a rich source of data. It registers the names of the victims, the date of the murders, the identity of the perpetrators, and the reasons they were killed (according to police reports, confessions of the perpetrators, and statements of the friends and families of the victims). I read the stories of 3349 women by clicking on each name listed on the Platform's website since 2008. Platform members from all over Turkey have contributed to creating the femicide database. In the organization's early years, this was achieved by analyzing all national newspapers and television news about femicides. Since 2017, the Platform has been present in each of the country's 81 cities. Every case of femicide in their local areas is reported to the headquarters, which is not always covered by the national media. In doing so, they try to collect as much transparent data as possible.

**FIGURE 4:**
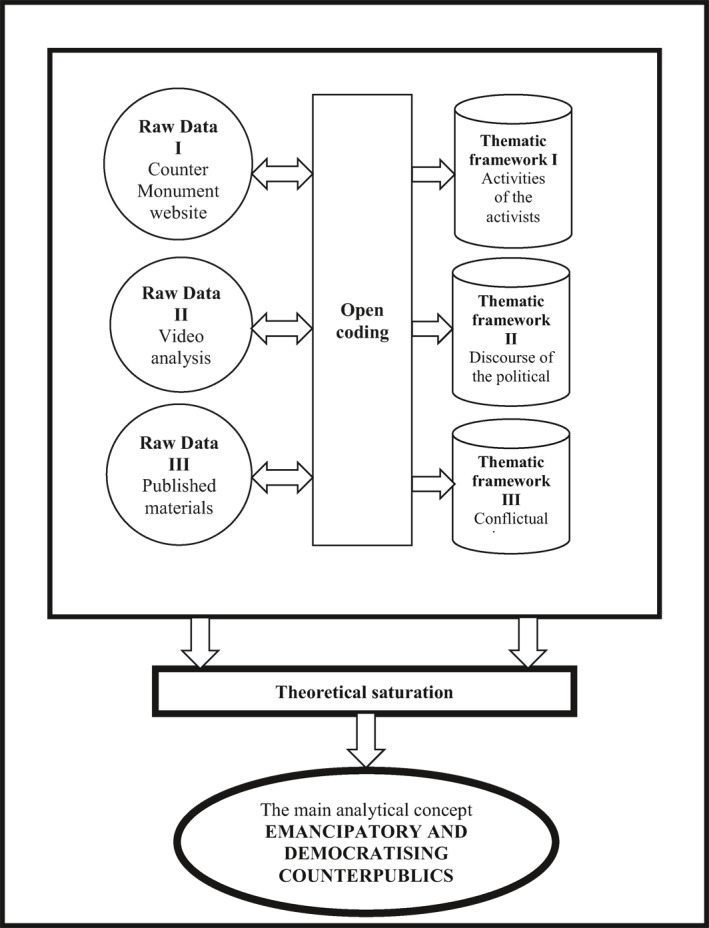
The coding process of raw data and the emergence of three thematic frameworks until obtaining theoretical saturation and the main analytical concept

After an inductive analysis of the Turkish women's social movement campaign, I studied 57 publications on the Platform's website (kadincinayetlerinidurduracagiz.net) and watched 37 videos from 5 to 77 min. Using grounded theory as a methodological technique in data analysis resulted in three thematic frameworks: (i) the activities of the activists, (ii) the discourse of the ruling political class; and (iii) the issues where conflict between the activists and the ruling political class occurs. Since the scope of the data was quite large and included written, visual, and media sources, the three frameworks assisted me in analyzing the data empirically. This was an appropriate strategy as I began my study to understand how women struggle against femicide rather than starting with a central research question. I used open coding to collect and analyze data. I processed 3349 femicide cases, 37,662 words, and 814 min of video footage as part of three separate raw data sets. I repeated the data analyses until I reached theoretical saturation and established the main concept and argument based on the analyses of the three main datasets consulted: (i) Monument Counter; (ii) 37 videos; and (iii) 57 published materials (see Figure 4).

In my second level of analysis, I found that the references to the past in the data and the use of victims and their stories in the digital space were frequently employed by the activists. At the end of the second round of analysis, I coined the term emancipatory and democratizing counterpublics to identify the relationship between femicide cases, the narratives of activists, and women's resistance in the digital space. The process of archiving femicide, generating new knowledge on femicide, and disseminating information on femicide cases and the reasons for it creates a collective memory and constitutes a political and legal intervention to reject the cultural norms of the political sphere and resist the epistemic injustice experienced by women.

There were challenges in collecting data based on reading the stories of women who had been murdered by men. As I read each story, I found myself in an emotionally difficult and stressful state, so I had to interrupt data collection from time to time when that stress became unbearable. These stories were so tragic that it was impossible not to think of the three children of a young woman who were sent to an orphanage after their mother was killed in front of their eyes. I had to read one similar story after another, which caused stress and emotional fragility in conducting my research and in my efforts to continue other activities in my daily life. This significantly prolonged the process of data collection and analysis, which interfered with my schedule for completing this research project. However, these breaks, which ranged from a few weeks to a few months, helped me cope with my emotional fragility and complete the research project. Even though the data of the murdered women and their stories are publicly available, I kept two main ethical standards in mind when presenting sensitive data. First, I avoided using the names of the children, although these were also publicly available. Secondly, I deliberately did not include visual sources in order to protect the victims and their acquaintances. The Platform's website prohibits the use of visual sources of murdered women. However, when the entry was made to recount the stories of the murdered women, local or national media news were mostly used as evidence.

Based on the data that I have collected, I argue that emancipatory and democratizing counterpublics consist of two main pillars: (i) the creation of a collective memory through the digital archiving of information about the murder of women through the Monument Counter, and (ii) informing the public and community members about the causes and grave consequences of femicide that contradict the views of hegemonic authority and the information that hegemonic authority disseminates to the public concerning the reasons for femicide and the role of women in society. These two pillars function in a harmonious mode in which the use of digital space and media technologies helps activists commemorate the murdered women, inform the public about their stories, manifest individual trauma, create a collective memory, and mobilize more people. The emancipatory and democratizing counterpublics show how the Turkish feminist movement can be transformative by challenging not only the policies of the ruling government regarding women's rights, but also the epistemic injustice that has increased its hold on women. The innovative methods developed by women to generate new knowledge, use digital space to disseminate it, and garner moral and public support demonstrate the importance of examining these methods of activists, which are critical for preventing femicide and challenging hegemonic authority.

## 4 CHALLENGING EPISTEMIC INJUSTICE AND MALE VIOLENCE

Epistemic injustice restricts women's capacity to expose the injustices to which they are subjected, and the growth of epistemic injustice creates a collective trauma for women who read in newspapers and watch on television almost daily reports of women being murdered by men. This is why activists' primary strategies for establishing counterpublics are to challenge epistemic injustice by compiling new data on murdered women, utilizing a new type of knowledge system to educate the public about the vital dangers women face, and connecting women from all walks of life via digital space. For members of the Platform, the assassination of Özgecan Aslan in 2015 galvanized them to take to the streets, make their struggle visible, and communicate their message to the masses.

The murder of Özgecan Aslan in 2015 represents a milestone for the Turkish feminist movement, as activists found fertile ground to draw attention to their struggle following a massive public backlash across the country. Aslan was a 19‐year‐old university student who resisted an attempted rape by the driver of the minibus that she was traveling home in. When she was the last passenger, the driver deviated from his regular route and stopped the minibus in a remote forested area. Aslan then fought back against the driver with pepper spray from her bag. However, the driver stabbed her several times until she died. After killing her, he called his father and a friend for help. The three men first cut off Aslan's fingers in an attempt to destroy physical evidence, as she had scratched the driver's face while she resisted his attempted rape. After removing her fingers, the three men then burned the body (Özgecan Aslan Cinayeti, 2015).

As the story of Aslan's murder continued to unfold on television and in the newspapers, public outrage in Turkey grew rapidly, so much so that the reaction was similar to that of the infamous gang rape in Delhi in 2012, when six men on a bus raped and beat up a 23‐year‐old physiotherapy intern who was on her way home. In Turkey, thousands of people took to the streets to protest, and nearly half a million women shared the Twitter hashtag “#*sendeanlat*” (“You must speak too”) over the course of 2 days to share their stories of harassment on Twitter. More than half a million people signed an online petition to demand more serious methods of fighting crime and harsher punishments for the perpetrators of women's murders (Woman's murder, 2015). Aslan's killers were finally brought to justice. All three men received prison sentences without the possibility of parole. The minibus driver, Ahmet Suphi Altındöken, was described as having committed:

‘A murder with monstrous instinct and torture, a murder with the motive of hiding a crime or evading capture, a murder due to the frustration caused by the inability to commit another crime, an attempt at sexual assault and deprivation of personal liberty with sexual motives’ (Özgecan Aslan davasinda karar, 2015).

Cammaerts (2015, p. 1031) states that the use of social media allows activists to more easily “self‐mediate” their grievances and significantly increases the ability to transmit both textual and visual discourses which assist marginalized groups to circumvent governmental and market controls. Furthermore, social media may play an important role in gaining allies who will support activists. Allies can provide strategic support for social movements to achieve their goals more effectively (Cayli, 2017; della Porta & Parks, 2013; Meyer & Staggenborg, 1998; Rucht, 2004). In our case, social media played a significant role in mobilizing people against femicide by gaining the support of elites. The Platform has a number of elite allies, ranging from opposition parties to leading popular writers and musicians (Eşitlik için, 2021). The main opposition party, the CHP, as well as minor opposition parties such as the HDP and TIP also support the Platform (Yavuz, 2021). The Platform has important allies in universities, such as the Universite Kadin Meclisleri (University Women's Assemblies). In addition, other public allies at universities demonstrate their support through university clubs, such as the Kadin Haklari Klubu (Women's Rights Club), whose members are also affiliated with the Platform.

The public's reaction to the murder expanded thanks to the mobilization efforts of activists from the Platform on social media, and the use of digital space played a crucial role in the court's verdict. However, dozens of women have been murdered but still have not received such a massive public response as the Aslan case. Consequently, members of the Platform seek legal changes by explicitly recognizing “femicide” in the Turkish Penal Code in order to prevent sentence reductions, which can be found in their online campaigns. The Platform claims that these sentence reductions lead to femicides continuing to be committed because perpetrators assume they will receive a sentence reduction, a sentence deferment, or not be punished with a severe life sentence. Using digital space is a strategic way to increase women's visibility online and ensure that victims' voices are heard when the mainstream media also reports these stories. The Platform's activists aim to use digital outreach effectively through Twitter and collaborate with other online media platforms to create a collective sphere of activism to stop femicide.

Erdoğan, the current Turkish president, has caused concern among Platform members because many of his speeches have raised obstacles to the emancipation of women. For example, he called on women to have at least three children, stating that “a woman who rejects motherhood, who refrains from being around the house, however successful her working life is, is deficient, is incomplete” (Turkish president says 2016). The Platform's activists first condemned this statement on their website and soon announced protests in Istanbul and Ankara. On behalf of the Platform, Gülsüm Önal asserted that such a statement violates human rights and the Turkish constitution. Önal added that the Turkish Constitution explicitly prohibits discrimination based on gender: “When the person who has the highest political position in the country makes such a statement, the state officials in the lower ranks, for example, in law enforcement, judiciary, and health services, start copying the same behavior” (Kadin Haklari Savunuculari, 2016). Erdogan's statement may very well fit into the concept of “the speaking state”, which defines the “proper” woman and contributes to the dynamics that lead to femicide (Atuk, 2020).

A group of women from the Platform's Eskişehir branch expressed their opposition to the discourse of politicians, violence against women, and violence against the LGBTQ+ community (Eskişehir'de Kadın Cinayetlerini Durduracağız, 2016). Their protest was especially important after the brutal murder of Hande Kader, an LGBTQ+ activist and sex worker. Kader was murdered by her clients, who later burned her body so that it was barely recognizable when police found it in one of Istanbul's forests (Hundreds protest over murder 2016). Dozens of protests were organized for Kader, and the Platform was one of the leading organizations in mobilizing people through the use of digital space. The Platform's activists also support the Saturday Mothers, a well‐known group whose members, mainly Kurdish mothers, have had their sons taken from their homes for interrogation and never returned. Since 1995, they have gathered every Saturday to protest political violence and enforced disappearances in Turkey in the 1980 and 1990s. They protest silently while displaying photographs of their loved ones. Platform activists also support Pride Month and assert that transgender murders are political murders (Kadinlar Ankara'dan Seslendi, 2018). Participation in these events is organized through digital communication channels and the exhibition of photographs. In addition, videos are instrumentalized in mobilizing more people. Platform members take new initiatives with activists from other stigmatized communities by using digital methods and online media. Thus, they create a network of solidarity with the survivors of other vulnerable groups seeking justice.

Seda Bayraktar, the leader of the Platform in Bolu (a city in northern Turkey), made a statement after a young nurse, Ayşegül Terziye, was harassed by a man on a bus because she was wearing a short dress. After kicking her in the face, the man shouted, “Those who wear shorts must die!” (Young nurse, 2016). The incident created a shockwave among the Platform members. The activists first published Terziye's story on their website and then organized a series of protests in different cities. A statement condemning the incident was published on the organization's website. Bayraktar, together with her colleagues from Bolu, protested against this harassment. In her statement, she said:

‘People who share common ground with ISIS militants and target secularism react harshly to the decisions we make for our own lives. In fact, secularism is the fundamental sphere in which we can defend the civil rights that we have earned through arduous struggle. For us, secularism represents the colourful dresses of the women who tore their clothes apart after being liberated from ISIS.’ (Kadın Cinayetlerini Durduracağız, 2016).

In court, the perpetrator who had harassed Terziye on the bus defended himself by saying: “I acted according to Islamic law (shari'a)”. The Platform issued another statement after the court hearing, noting that the irresponsible attitude of politicians and their tacit support of perpetrators lead to the undermining of the principle of equality of the justice system guaranteed by the constitution. Platform members protested the incident in various cities, mobilized people to join the protest through social media and released a statement and sent it to the mainstream media: “Women can wear whatever they want, walk whenever they want, and go out at any time of night. If there is no state protecting them, we are here. We will change this dispiriting and dark situation” (Puslu karanlık havayı dağıtacağız, 2016). Platform members regularly organize events across the country to draw attention to the waning influence of women's rights in the country. Terziye's harassment enraged activists, who mobilized Platform members across cities via social media to protest the harassment on different dates during the same week. The Edirne branch organized a social protest in the city in 2016 for this reason. In this protest, spokesperson Özgün Derin stated:

‘Women are killed or tortured simply because they want to divorce or make their own decisions about their lives. We are in an authoritarian era in which we have been targeted more frequently when we wear shorts (indicating mini‐shorts)’ (Kadinlar laiklik icin direniyor, 2016).

Platform members have established *Üniversite Kadın Meclisleri* (University Women's Assemblies) across the country and have representatives at each university to organize and mobilize more women. Digital communication plays an important role in organizing unions and recruiting volunteers. This allows members to educate young people about the causes of femicide. Activists who join the Platform include doctors, psychologists, lawyers, and other professionals who investigate the mysterious deaths of women, often recorded as suicides. They also offer help and support to women when they face threats or sexual assault. Digital space plays a key role in promoting these activities by effectively using the website, media, images, and videos in the context of relevant policy changes to save women's lives.

Walby (2020, p. 413) contested the tendency to simplify or combine the numerous elements of gender into the concept of the family and emphasized the need to extend the critical thinking of gendered regimes within four domains, including the economy, politics, civil society, and violence. In addition, Hollander (2013) emphasized the significance of accountability in the maintenance or destruction of gendered regimes. Taking into account the arguments of Walby (2020) and Hollander (2013) on gendered regimes, the use of digital space by the members of the Platform transforms individual trauma into collective trauma in the public sphere. In this way, activists mobilize more individuals and politicize the public sphere, so they do not limit the gendered regime to the family concept alone. They also attempt to destroy the current gendered regime by commemorating the murdered women and drawing attention to discrimination against vulnerable groups such as LGBTQ+ people, refugees, and Kurdish women. The heterogeneous nature of the movement has been evident every 8th of March when the LGBTQ+ community, Kurdish women, and refugee women walked shoulder‐to‐shoulder with Platform members on the streets of different cities across Turkey on International Women's Day (Magid, 2018). The methods used in creating emancipatory and democratizing counterpublics manifest the concerns of the Turkish feminist movement through digital space and challenge the cultural norms dictated by the state. In doing so, the use of digital tools and new data created by women not only provides a platform for collective memory but also exposes the discriminatory discourse of politicians and concretizes their protest through collective resistance in which they bring together other vulnerable groups for their cause, along with the cultural values based on justice and equality.

The activists are trying to put pressure on lawmakers to amend the Law for Protection of Family and Prevention of Violence against Women Act No. 6282, which includes “femicide” as a legal term in the Turkish Penal Code. Furthermore, the Platform wants to punish femicide with “aggravated life imprisonment” and calls for “the annulment of continuing abatements” (For English, 2016). All of the Platform's proposals and amendments regarding its demands to change the Turkish Penal Code refer to the Istanbul Convention (2011).^3^ The inclusive and effective legal measures of the Convention make it the second most important international convention for the prevention of violence against women, after the Convention on the Elimination of All Forms of Discrimination against Women, signed in New York in 1979. However, unlike the New York Convention, the Istanbul Convention is a more effective and comprehensive document that aims to combat violence against women by preventing domestic violence, protecting victims and prosecuting offenders. It has been in force since August 1, 2014, and Turkey was the first signatory country among 44 other countries in January 2017 (Chart of signatures and ratifications of Treaty 210 2017). States ratifying the Istanbul Convention are required to criminalize a range of offenses, including psychological violence (Art. 33), stalking (Art. 34), physical violence (Art. 35), sexual violence, including rape, which explicitly includes all non‐consensual sexual acts with a person (Art. 36), forced marriage (Art. 37), female genital mutilation (Art. 38), and forced abortion and forced sterilization (Art. 39).

**FIGURE 5:**
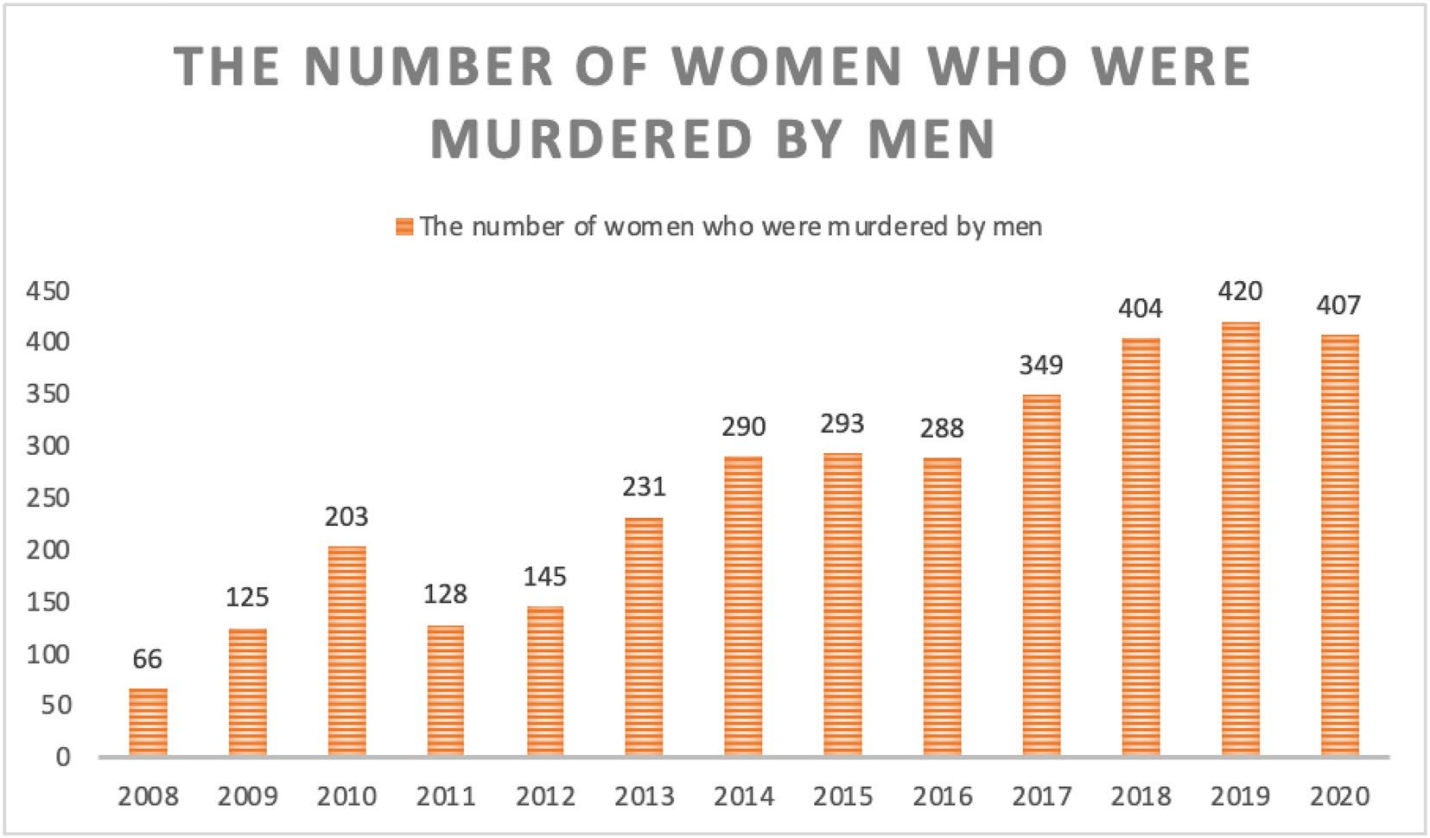
The absolute number of women murdered by men in Turkey per year from 2008 to 2021. Data extracted from the Platform's website. (Figure was created by the author)

The importance of the Istanbul Convention and its outcomes can also be seen in the decrease in the number of femicides. Members assume that this is due to the deterrent effect of the Convention (see Figure 5). The Platform has made this assumption because it believes that the widespread news in the media had a deterrent effect on male offenders when the Convention was signed in 2011 and had an immediate effect in the same year. However, this does not fully explain the gradual increase in the following years. There could be three possible reasons for this increase: (i) the conservative political discourse used by the ruling party over time, (ii) the insufficient adaptation of the Istanbul Convention when survivors faced violence and intimidation and told law enforcement agencies about their traumatic experiences; and (iii) the increasing mobilization efforts of the Platform members, which helped them to better organize and record cases of femicide across the country.

Although it is difficult to prove the decrease in the number of femicides based on the impact of the Istanbul Convention, it can be argued that it has been one of the most effective conventions to ensure women's social and legal rights. From 2010 to 2019, the number of femicide cases surged with the rise of religious discourse and references to Islamic sources that shaped policy by treating women based on the ruling party's interpretation of cultural norms and the dictation of these norms in society through the media and policy. These socio‐cultural dictates have taken root in police stations, criminal justice systems, and social services. Bureaucratic authority is responsible for enforcing the laws protecting women, and this enforcement has not always been fast enough to protect women who need help in the face of abuse or threats by men. This was one of the reasons why the Platform members organized a series of social protests for the effective implementation of the Istanbul Convention. As Kav put it, “if the Convention had been enforced correctly, we would not be talking about femicide cases today” (Pınar Gültekin, 2020). The significance of the Convention is made clear by this criticism from the Platform's Director. More importantly, her perspective illustrates why signing international conventions may not bring real changes to the lives of vulnerable groups. Through its effective implementation, women expected that the necessary steps would be taken to make the Istanbul Convention meaningful for their lives. However, Erdoğan withdrew from the Convention through a presidential decree announced on March 20, 2021.

The withdrawal from the Istanbul Convention has wreaked havoc on millions of women. Previously, women at least had a legal basis to put pressure on law enforcement agencies and judges to justify their claims against male violence by invoking the articles of the Convention, although such claims were usually ignored. As a result of the withdrawal from the Convention, women have lost their basic legal protection. Legal experts on constitutional law argue that the withdrawal from the Istanbul Convention with an executive decree signed by the President is not valid according to the principle of parallelism in authority and procedure in public law, which signifies that only the institution that is authorized to make a law can abandon the same law. As the Istanbul Convention was approved by the Turkish Grand National Assembly, it can be abandoned only by the same institution and not by a presidential decree. According to constitutional law experts, the President's executive decree withdrawing from the Istanbul Convention is invalid under the principle of parallelism in authority and procedure in public law, which states that only the institution authorized to make a law may repeal it. Due to the fact that the Istanbul Convention was ratified by the Turkish Grand National Assembly, it can be revoked only by that body, not by presidential decree (Istanbul sözleşmesi'nin feshi, 2020). The opposition parties, the CHP and the HDP, protested the withdrawal from the Istanbul Convention, and their leaders pledged to ratify it again once they gained power to rule the country (TBMM'de CHP ile HDP'den İstanbul Sözleşmesi protestosu, 2021).

The decision to withdraw from the Convention was unpopular but gained momentum when conservative religious groups supporting the ruling government portrayed the Convention as destructive to Turkish society, claiming that it damaged family values by encouraging women to divorce and made young people accept LGBT communities as if they were “normal” (Koylu, 2020). Millions of people used the hashtag “#istanbulsozlesmesiyasatir” (“The Istanbul Convention helps women stay alive”) on Twitter the same night and the following days after the withdrawal, thanks to the efforts of Platform members (Kadınlar İstanbul Sözleşmesi için sokakta, 2021). The influence of heteronormativity on society derives not only from repressive social forces that impose their standards on weaker groups, but also from legal developments that make it more difficult for women and non‐binary people to be free.

In authoritarian regimes, when the state exercises de facto monopoly power over the media, the public sphere mirrors hegemonic discourse to a great extent (Dukalskisk, 2017). In this respect, online media may pose significant risks to the sustainability of oppressive rulers by mobilizing people to take to the streets to protest and express their strong opposition to authoritarian regimes (Ruijigrok, 2016). By leveraging protest and digital action, online networks play a critical role in establishing counterpublics and stimulating social change (Earl, 2013). For example, in Russia, the information offered to the public via official media sources is frequently one‐sided and filtered via layers of self‐censorship, even though the institutional opposition exists but is not able to provide substantive alternative information (March, 2009). Other activists used online platforms to empower subaltern counterpublics and influence public opinion on issues ranging from racial justice to queer resistance (Jansen, 2017; Kuo, 2018; Lo, 2022; Vrikki & Malik, 2019). They facilitated counterpublics through the use of similar methods to expose injustices they encountered on online and offline platforms. Subaltern counterpublic groups frequently use social media to demonstrate their resistance and organize protest events in public spaces (Salmenniemi, 2019; Zeng, 2014).

The Platform's activists also employ similar tactics in other countries by expanding the capacity of counterpublics through online platforms. However, three critical factors distinguish Turkish activists from other struggles: (i) the generation of new data on femicide and its preservation on the online platform; (ii) the effective use of the online platform for mobilization and shaping public attitudes toward femicide by sharing the data with the public, giving voice to the victims and survivors, democratizing the public sphere and removing it from the influence of the current government's ideology; and (iii) the Platform's ability to sustain their mobilization, which has grown exponentially since their first struggles 15 years ago, making it the oldest and largest activist group fighting femicide through data collection, the use of digital platforms, and innovative dissemination methods to manifest their objectives and shape public discourse.

In other countries, the use of digital platforms against femicide has gained momentum in the last decade. For example, the Canadian Femicide Observatory for Justice and Accountability (CFOJA) was established in 2015 to mobilize people, exchange, and promote research and knowledge to prevent femicide and other forms of gender‐based killings in Canada (Dawson, 2018). Nonetheless, the Platform's members' facilitation of digital tools and tens of thousands of volunteer activists elevate it to a critical social movement whose fight against epistemic injustice and male violence is critical to millions of women's survival. Women bear the brunt of male violence in both developing and developed countries. However, in Turkey, as well as other authoritarian regimes and developing countries, women's vulnerability is exacerbated by a lack of access to fair justice, ineffective law enforcement protection, and economic precarity. This is why the Platform's activities and effective use of digital space create a space for addressing the severe impact of intersectional injustice, ensuring women's safety, increasing solidarity, and influencing public opinion about women's status and the injustices they face on a daily basis.

## 5 THE DIGITAL SPACE OF COUNTERPUBLICS AND THE DEMOCRATIZATION OF METHODS AGAINST EPISTEMIC INJUSTICE

Milan (2015) and Kaun (2016) show that the use of social media increases the political visibility of collective action in social movements. Media technologies shape social structures in an accelerated way (Rosa, 2003). Similarly, Poell (2020, p. 609) focuses on the temporality of social protests using media, arguing that social protests largely fail to capture the attention of mainstream media when they are “covered from an episodic perspective, ignoring larger protest issues.” In this respect, the repoliticization of media infrastructures can play a major role in creating an effective communication channel (Kaun, 2017, p. 469). The examples I have given show that Platform activists not only politicize the public sphere but also democratize both the public sphere and data by creating a publicly accessible archive of murdered women and disseminating knowledge through digital channels in order to mobilize more people and broaden the moral base of their struggle. Activists spread knowledge about femicide and advocate for the basic human rights of other vulnerable groups in the country, such as the LGBTQ+ community, refugees, and ethnic female minorities. Thanks to the growth of digital space and tools, their alliance extends the emancipatory and democratizing counterpublics.

The form and dissemination of knowledge are critical for understanding the limits of epistemological injustice and how these limits can be overcome through women's innovative initiatives that result in their emancipation, society's democratization, and access to sensitive data. Activists' struggle against injustice is particularly critical in authoritarian regimes that restrict access to data and attempt to shape public opinion in accordance with their own political and cultural ideologies. On the one hand, digital space provides a platform for vulnerable groups and democratizes the public sphere. On the other hand, by generating widespread public support for their cause, their resistance efforts toward emancipation gain momentum. Even though this does not guarantee ultimate victory over the authoritarian regime, given the withdrawal from the Istanbul Convention, the Platform's activists work to increase public legitimacy, putting authoritarian regimes' survival at risk as moral and electoral support for the ruling government dwindles in recent polls. Konda's face‐to‐face poll, conducted in 2020 with 3569 people in 206 neighborhoods and villages in 110 districts of 32 Turkish cities, revealed several significant findings for the anti‐femicide campaign. Only 16% of respondents support Turkey's withdrawal from the Istanbul Convention, while 84% oppose government policies and believe Turkey should sign and implement the Istanbul Convention. Among the most striking findings is the evolution of society's justifications for gender‐based violence and femicide over time. For example, in 2015, 80% of women agreed with the statement “women must pay attention to their clothes and how they dress,” but only 32% agreed in 2020 (Konda, 2020). This highlights the critical importance and impact of digital campaigns, as well as the mobilization efforts of all women who have unwaveringly contributed to the creation of emancipatory and democratizing counterpublics. As a result of Platform members' digital campaigns organizing protests in physical spaces, their visibility in these public spheres has increased. In accordance with this argument, Moghadam (2013, p. 204) stated that the internet has not eliminated the need for physical venues of recruitment and action, but it has enabled the emergence of virtual activism and the more effective use of public spheres.

Kidd and McIntosh (2016) note that the role of social media in political change through online activism has produced two opposing camps: (i) an optimistic approach that claims that the revolution will be tweeted, and (ii) a pessimistic approach that claims that social media can actually mitigate positive change. Ultimately, they argue that we need to develop an ambivalent approach by acknowledging the claims of both camps so that it would be more reasonable to make judgments based on evidence and consider that positive change may be possible through social media. However, this would also be a difficult task. Data from this study shows that feminist activists play a crucial role in shaping the conventional discourse and challenging political and legal authorities. The use of digital space by Turkish women is more focused on generating original data and using this data to inform the public, gain public support, politicize the public sphere, and challenge the social and cultural norms of the ruling authority. Turkish women use digital space innovatively by generating new data and disseminating it, which democratizes the methods to defy epistemic injustice and critically helps to save the lives of millions of women.

By disseminating knowledge based on the collected data, activists develop solutions to the challenges women and other stigmatized groups face in their everyday lives. In this context, it is important to note the argument of Fenton (2008, p. 238), who states that “claims for the extension and reinvention of activism must be considered in the context of the material social and political world of inequality, injustice, and corporate dominance. If it is true that a global civil society is developing on the web, it is one that is segmented by interest and structured by inequality”. The activists' struggles in Turkey have created a collective memory that is a tragic result of the murder of women. However, they have also transformed their collective solidarity into concrete political action to fight epistemic injustice and male violence. Their struggle in digital space democratizes the public sphere by producing new data and disseminating new forms of knowledge. The work of feminist activists defies the moral basis of hegemonic political authority that defines women within the boundaries of patriarchal and religious values. Nonetheless, this attempt at democratization by the activists leads to conflict with the hegemonic authorities.

**FIGURE 6:**
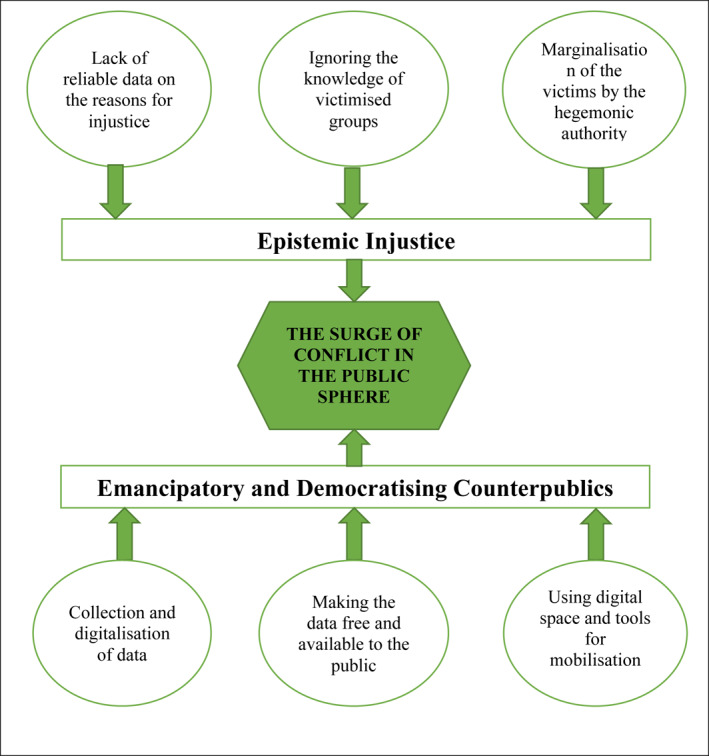
The figure explaining the surging conflict through the relationship between the outcomes of epistemic injustice and emancipatory and democratizing counterpublics

The withdrawal from the Istanbul Convention implies two outcomes: (i) the long struggle of Turkish women does not guarantee that they can achieve their main objectives in a short period of time; and (ii) their effective use of digital space to prevent femicide, create new forms of knowledge and disseminate it, as well as their efforts to mobilize people, and politicize the public sphere draw the furious reaction of the authoritarian regime against the movement. Therefore, social conflict surges between the hegemonic authority and the actors of counterpublics. The figure of the public sphere of conflict explains how social conflict surges when activists employ three important methods: (i) collecting and digitalizing data, (ii) making the data free and available to the public, and (iii) using digital space and digital tools for mobilization. The epistemic injustice targeted by women is composed of three key factors: (i) the lack of data on murdered women, (ii) the ignoring of the knowledge of victimized groups; and (iii) the marginalization of victims by hegemonic authority based on its own moral standards (see Figure 6).

There are two points worth emphasizing. To begin with, not all counterpublics contribute to the democratization of the public sphere. Given the rise of right‐wing political groups in recent years in Europe and North America, they are also counterpublic forces, but their opposition to the current public domain is based on their race or political ideology being superior to others, which is diametrically opposed to the counterpublics created by Platform activists. This is why emancipatory and democratic counterpublics seek not only to undermine the ruling authority but also to expand people's rights based on principles of justice and equality. In addition to attempting to change policies that directly affected their lives, transnational feminist networks also sought to influence “cultural norms and broader societal transformation” (Moghadam, 2013, p. 167). In this regard, the objectives of the Platform are well aligned with the goals and scope of transnational feminist networks. Second, the escalation of public conflict and the struggle of resisting groups for their rights under an authoritarian regime may aggravate their situation, as evidenced by Turkey's withdrawal from the Istanbul Convention. Based on a comparative review of anti‐authoritarian social movements, Tufekci (2017) concluded that digital media and digital instruments in the 21st century gave significant support for the initial success of social movements and facilitated equitable participation. However, this also poses additional risks to the sustainability of such movements, especially in the absence of leadership and a clear and consistent agenda setting. Our study also confirms that, in the short term, the surge of conflict in the public sphere poses risks to resisting groups. In the long run, however, their efforts provide hope for democratizing the public sphere and shaping public opinion, transforming them into social forces of change in society, both in terms of moral codes and political choices, posing greater risks to authoritarian regimes that risk losing public legitimacy.

## 6 CONCLUDING REMARKS

This study has sought to explore how feminist activists strive to replace the religious and conservative cultural values of the ruling political sphere with principles of equality and justice by using digital space and digital tools. The principal causes of epistemic injustice are based on the lack of reliable data on murdered women; the power of ruling actors limiting women's ability to articulate their grievances and injustices; and the moral standards imposed on women, obliging them to comply with those standards. Without transparent data and instruments to address the consequences of femicide and the myriad causes, epistemic injustice exacerbated women's vulnerability. Nonetheless, women have not remained silent, and their innovative anti‐femicide policies seek to eradicate epistemic injustice by establishing emancipatory and democratizing counterpublics. Digital methods and digital space have been used by a number of feminist organizations across the world to mobilize people for their cause and challenge the social and cultural norms of hegemonic authority for the last 2 decades (Berg & Carbin, 2018; Clark‐Parsons, 2022). Similarly, the struggle of Turkish women demonstrates that the rapid digitalization process offers new opportunities for mobilization and social transformation. This study revealed, however, that substantial use of digital tools and support from elite allies may not be strong enough to resist the policies of an authoritarian government. As evidenced by the withdrawal from the Istanbul Convention, such opposition to the political authorities can even result in a diminution of women's rights.

Authoritarian governments pose risks to resisting groups by preserving the status quo through the creation of severe obstacles for those groups within a culture deeply colored by policing and punishment regimes. Hence, changing a society's cultural values requires altering the ruling hegemonic authority, the political sphere that regulates social forces, and the legal spheres of social life. This challenging reality, therefore, motivates and encourages the members of the Platform to take concrete actions to mobilize more people by using digital space. I have endeavored to show that women's struggles are not only a protest against the violation of the fundamental right to survival but also a transformative force to defend and guarantee the rights and safety of vulnerable groups in the country, considering their support for LGBTQ+ people, Kurdish women, refugees, and ethnic minority women. The use of digital space thus democratizes our methods of addressing epistemic injustice by creating new data and disseminating it online.

The activists want to gain public support for most of the policy changes they propose, which could force either the current government or future governments to make the necessary legal changes to prevent femicide. However, these changes also require the elimination of gender inequality and injustice. This is why the women's struggle is more than a struggle to prevent femicide; it points to a change in hegemonic authority. Individuals' decisions on whether and how to join a social movement are influenced by external political circumstances, mainstream political institutions' opportunities for action, organizational resources and commitments, and emotional and cognitive elements (Meyer, 2002, p. 10). Law and order factions of the political elite may be less effective if other factions of the political elite encourage mobilization and collective action at the same time (Koopmans, 1999, p. 105). The Platform's methods for establishing emancipatory and democratizing counterpublics are a powerful and persuasive way of instrumentalizing digital space to aid women in their struggle for survival. Nevertheless, it also stimulates the reaction of authoritarian government bodies, which creates a higher risk of failure to achieve the objectives of the Platform. As this study has attempted to demonstrate, however, such a struggle against authoritarian regimes may lead to a reversal of rights. Although the Turkish feminist movement offers women a promising future in the long term, women's rights in the country have declined in recent years. This compels us to reconsider the significance of determining both short‐term and long‐term strategies for social movement organizations. Organizers and leaders of social movements may benefit from designing short‐term and long‐term strategies that enable them to produce effective policies with immediate effects on people's lives. By doing so, they not only pose a long‐term existential threat to authoritarian regimes, but also devise a policy agenda that significantly improves the lives of oppressed groups.

The withdrawal from the Istanbul Convention is the high price of standing up to authoritarian rule in the short term. And this is why activists work overtime to emancipate and democratize the public sphere, particularly by gaining the trust of the masses. Increased activism in the aftermath of the Convention's withdrawal as well as recent polls demonstrate that in authoritarian regimes, emancipatory and democratic counterpublics must consider temporality and set long‐term goals to influence elections and create the potential for democratic regime change.

The emancipatory and democratizing counterpublics can be applied by researchers in other contexts to study how social movements under authoritarian regimes are mobilized to fight for justice. Highly sensitive research topics can pose risks to the researcher's reflexive capacity and emotional resilience. Indeed, I experienced extreme stress reading the stories of hundreds of murdered women, the reasons they were murdered, and the grim consequences when justice was not served. For this reason, I recommend that future researchers studying traumatic experiences take a break from data collection when the stress becomes unbearable. Furthermore, because this study is limited to digital space, there are challenges in accessing thick descriptions crucial to exploring activists' everyday experiences through ethnographic and in situ observations. If researchers aim to use the emancipatory and democratizing counterpublics concept to explore different cases through ethnographic data collected in physical space, this may help us to explore the victimization process more systematically, which is not always possible through the use of digital ethnography. This could also open up new opportunities for researchers to identify women's experiences and the unjust policies imposed on them through political and legal channels in both liberal and illiberal democracies.

Digital space allows researchers to analyze the responses of vulnerable groups and the socio‐cultural positions of less powerful groups in society when this is not possible by other means. This study shows that women's use of digital space takes on particular significance in defying hegemonic power relations in authoritarian regimes while also posing serious perils to the rights of women. I have endeavored to show that the Platform's activists break the silence on femicide and expose the criminal context of cultural norms in digital space using digital methods when the hegemonic political sphere limits their use of physical space. The activities of Platform members in creating new knowledge and disseminating it through digital channels contribute to understandings of the sociology of epistemic injustice and the challenges of using digital space and instruments in authoritarian regimes. This study suggests that if the short‐ and long‐term strategies of social movement organizations are determined, and the risks of mobilizing against authoritarian governments are effectively addressed, media technologies and digital tools can play an important role in the lives of vulnerable groups. As a result, millions of oppressed groups fighting for their fundamental rights will facilitate not only the development of a social and legal system that safeguards the rights of vulnerable groups but also the transition from autocratic to democratic political regimes.

## CONFLICT OF INTEREST

There is no conflict of interest disclosure to be noted.

## ETHICS STATEMENT

The research project received ethics approval from the University of Derby on July 19, 2016.

## PATIENT CONSENT STATEMENT

The research project does not require patient consent statement.

## PERMISSION TO REPRODUCE MATERIAL FROM OTHER SOURCES

The research project does not require the permission to reproduce material from other sources.

## Data Availability

The data that support the findings of this study are available from the corresponding author upon reasonable request.

## ENDNOTES

^1^
The organization's official name in Turkish is Kadın Cinayetlerini Durduracağız Platformu.

^2^
The website can be accessed via www.anitsayac.com.

^3^
The official name of the Istanbul Convention is “Convention on preventing and combating violence against women and domestic violence”. It was first signed on May 11, 2011.
